# Supplementary material for: Myofibril diameter is set by a finely tuned mechanism of protein oligomerization in Drosophila
Source: eLife. 2019 Nov 20;8:e50496. doi: 10.7554/eLife.50496 (PMC6910826; doi:10.7554/eLife.50496)
Supplement: Supplementary file 1. [file elife-50496-supp1.docx]

Detailed genotypes for all figures

Figure 1.

- *Act88F-Gal4, Zasp52-mCherry, UAS-LacZ*
- *Act88F-Gal4, Zasp52-mCherry, UAS-GFP-Zasp52-PR*
- *Act88F-Gal4, Zasp52-mCherry, UAS-Flag-Zasp52-PP*
- *Act88F-Gal4, Zasp52-mCherry, UAS-Flag-Zasp52-PR∆ZM*
- *Act88F-Gal4, Zasp52-mCherry, UAS-Flag-Zasp52-PR∆PDZ*
- *Act88F-Gal4, Zasp52-mCherry, UAS-Zasp66-PK-Flag*
- *Act88F-Gal4, Zasp52-mCherry, UAS-Zasp67-PD-Flag*
- *Act88F-Gal4, Zasp52-mCherry, UAS-GFP-Zasp52-PK*
- *UH3-Gal4, Zasp52-mCherry, UAS-LacZ*
- *UH3-Gal4, Zasp52-mCherry, UAS-GFP-Zasp52-PR*

Figure 3 A-D.

- *Zasp66-GFP/+*
- *Zasp66-GFP/+; Zasp52^MI02988^/Df(2R)BSC427*
- *Zasp66-GFP/+; Zasp52^MI02988^/Df(2R)BSC427*

Figure 3 E-H.

- *Act88F-Gal4, UAS-Zasp52-PK-NYPF, UAS-Zasp52-PK-CYFP*
- *Act88F-Gal4, UAS-NYPF, UAS-Zasp52-PK-CYFP*
- *Act88F-Gal4, UAS-Zasp66-PH-NYPF, UAS-CYFP*
- *Act88F-Gal4, UAS-Zasp66-PH-NYPF, UAS-Zasp52-PK-CYFP*

Figure 5.

- *Zasp52-GFP*
- *Zasp67-GFP*
- *Zasp66-GFP*

Figure 6A-C.

- *Zasp67-GFP/+*
- *Zasp67-GFP/+; Zasp52^MI02988^/Df(2R)BSC427*
- *Zasp67-GFP/+; Zasp52^MI02988^/Df(2R)BSC427*

Figure 6D-G.

- *Act88F-Gal4, Zasp52-mCherry*
- *Act88F-Gal4, Zasp52-mCherry, UAS-Flag-Zasp52-PP*
- *Act88F-Gal4, Zasp52-mCherry, UAS-Flag-Zasp52-PP*143*
- *Act88F-Gal4, Zasp52-mCherry, UAS-Zasp66-PK-Flag*
- *Act88F-Gal4, Zasp52-mCherry, UAS-Zasp67-PD-Flag*

Figure 7A-E.

- *Zasp52-mCherry/+;*
- *Zasp52-mCherry/+; Zasp66 -/-*
- *Zasp52-mCherry/+; Zasp67 -/-*
- *Zasp52-mCherry/+; Zasp66 -/-, Zasp67 -/-*

Figure 7F-I

- *Act88F-Gal4; UAS-GFP-Zasp52-PR, UAS-LacZ*
- *Act88F-Gal4; UAS-GFP-Zasp52-PR, UAS-Flag-Zasp52-PP*
- *Act88F-Gal4; UAS-GFP-Zasp52-PR, UAS-Flag-Zasp52-PP*143*
- *Act88F-Gal4; UAS-GFP-Zasp52-PR, UAS-Zasp66-PK-Flag*
- *Act88F-Gal4; UAS-GFP-Zasp52-PR, UAS-Zasp67-PD-Flag*

Figure 1—figure supplement 2.

- *Act88F-Gal4; Zasp66-GFP/+*
- *Act88F-Gal4; UAS-Actn-KK; Zasp66-GFP/+*
- *Act88F-Gal4; UAS-GFP-Zasp52-PR, UAS-LacZ*
- *Act88F-Gal4; UAS-GFP-Zasp52-PR, UAS-Actn-KK*
- *Act88F-Gal4, Zasp52-mCherry, UAS-GFP-Zasp52-PR*
- *Act88F-Gal4, Zasp52-mCherry, UAS-Zasp52-PR-GFP-Flag*

Figure 2—figure supplement 1.

- *UAS-GFP; Mef2-Gal4, UAS-Flag-Zasp52-PK*
- *Zasp52-GFP; Mef2-Gal4, UAS-Flag-Zasp52-PK*

Figure 3—figure supplement 1.

- *Act88F-Gal4; UAS-Zasp66-PH-GFP*
- *Actn-GFP/+*
- *Actn -GFP/+; Zasp52^MI02988^/Df(2R)BSC427*
- *Actn -GFP/+; Zasp52^MI02988^/Df(2R)BSC427*

Figure 6—figure supplement 1.

- *Act88F-Gal4, Zasp52-mCherry*
- *Act88F-Gal4, Zasp52-mCherry, UAS-Flag-Zasp52-PP*
- *Act88F-Gal4, Zasp52-mCherry, UAS-Flag-Zasp52-PP*143*
- *Act88F-Gal4, Zasp52-mCherry, UAS-Zasp66-PK-Flag*
- *Act88F-Gal4, Zasp52-mCherry, UAS-Zasp67-PD-Flag*
- *Act88F-Gal4, Actn-GFP/+, UAS-LacZ*
- *Act88F-Gal4, Actn-GFP/+, UAS-GFP-Zasp52-PR*
- *Act88F-Gal4, Actn-GFP/+, UAS-Flag-Zasp52-PP*
- *Act88F-Gal4, Actn-GFP/+, UAS-Flag-Zasp52-PR∆ZM*
- *Act88F-Gal4, Actn-GFP/+, UAS-Flag-Zasp52-PR∆PDZ*
- *Act88F-Gal4, Actn-GFP/+, UAS-Zasp66-PK-Flag*
- *Act88F-Gal4, Actn-GFP/+, UAS-Zasp67-PD-Flag*
- *Act88F-Gal4, Actn-GFP/+, UAS-GFP-Zasp52-PK*
